# Editorial: Immunotherapy in Multiple Myeloma

**DOI:** 10.3389/fimmu.2019.01945

**Published:** 2019-08-14

**Authors:** Nicola Giuliani, Fabio Malavasi

**Affiliations:** ^1^Department of Medicine and Surgery, University of Parma, Parma, Italy; ^2^U.O. di Ematologia e Centro Trapianti Midollo Osseo, Azienda Ospedaliero-Universitaria di Parma, Parma, Italy; ^3^Department of Medical Science, University of Turin and “Fondazione Ricerca Molinette”, Turin, Italy

**Keywords:** multiple myeloma, immunotherapy, monoclonal Ab, CD38, immune checkpoints

## Honoring The Past

Multiple myeloma (MM) is a hematological malignancy characterized by a high tendency to relapse and to become drug resistant. In the past, melphalan was considered the standard of the treatment for MM patients (1). Following, the introduction of thalidomide and the proteasome inhibitor bortezomib leaded to a significant improvement of the survival of MM patients: however, none of them reached the cure of the disease. These drugs introduced the concept of the treatment of MM patients targeting not only the malignant clone but also the microenvironment (2). In addition, the introduction of lenalidomide, a thalidomide derivative, expanded this concept by focusing to the immune-microenvironment (2). More recently, the introduction of the monoclonal antibodies (mAbs) seem to change the paradigm of MM treatment, highlighting the possibility to cure MM patients by an immunotherapeutic approach (3).

Immunotherapy is part of a concept that goes back to the beginning of 1900. The “magic bullet” opened the way to the objective of having a tool able to selectively eliminate target cells and at the same to modulate the immune response in a beneficial way. It was necessary to wait for several decades and apparently for unrelated findings coming from different fields before having a picture able to frame the view outside a simple anecdotal hope. The objective was initially made possible by combining results from basic science and the availability of mAbs, a reagent made of a homogeneous population of immunoglobulins (Ig), the main difference from the conventional antisera. mAb is hence able to recognize only a single epitope on the molecular target.

The second key event derived from the identification of a vast number of soluble factors, which share the feature of transmitting signals in the context of similar or different cells (the interleukins). Using this new tools, Reinherz et al. generated a panel of mAbs specific for surface molecules located on the surface of human T lymphocytes (4). At the same time, Smith et al. made available IL-2, a cytokine which made possible to expand clones of normal T lymphocytes (5). Combining these approaches, Reinherz et al. (4) were able to define murine reagents specific for molecules present on all T lymphocytes, while other ones were limited to subsets of the same cells. Another set of mAbs recognized molecules only present in single lymphocytes (defined as idiotypic). These findings modified the simplistic dogma that mAbs were only able to bind the target antigen and to induce cell lysis. This lead to the definition of the concept of agonistic antibodies: the translational inference is that the engagement by a mAb of selected domain of a molecule may surrogate the effects induced by a natural ligand of the same receptor, even when the ligand was not known.

The definition of immune check points molecules lead to the preparation of panels of mAbs able to induce or brake immune responses, according to distinct medical needs. The concept of immune modulation was further completed by the identification of activator effector T and B cells, while other cell subsets produce effects going in opposite directions (Treg, Breg, and myeloid-derived suppressor cells are the most important). The considerations derived from the dissection of the main steps of immune cell defense were indirectly confirmed by studies conducted on the different strategies of immune escape of tumors. Indeed, it emerged that some tumors adopt escape or camouflage strategies, which implement genetic programs driving to metabolic reshaping, secretion of immunomodulatory cytokines, or generation of tolerogenic substances, among the others.

## Studying the Present

At the moment, available to the medical community there is a panel of therapeutic antibodies, which significantly modified the fate of some diseases, especially neoplasies of hematological origin. There is a general agreement that the therapeutic effects are prevalently obtained by means of antibody-dependent cellular cytotoxicity (ADCC), antibody-dependent cell-mediated phagocytosis (ADCP), and complement-dependent cytotoxicity (CDC), and concurrently by the induction of signals on cell effectors (6). These effects may be enriched by other functions exerted by the target molecule, such as the ability to lead to generation of substrates able to induce immunomodulation. This is the case when a molecule belongs to the family of ectoenzymes, which now encompasses almost 5% of the entire surface molecule express by leukocyte (6). Other mAbs were generated against soluble molecules produced by both MM cells and the bone microenvironment including sclerostin able to block MM-derived bone destruction and in turn MM progression.

In the treatment of MM, the Food and Drug Administration (FDA) approved daratumumab (DARA) and elotuzumab (Elo), two monoclonal IgG-k mAbs, specific for CD38 and SLAMF7 (signaling lymphocytic activation molecule F7), respectively. The approval was for the treatment of relapsed or refractory MM (RRMM) patients, in combination with lenalidomide and dexamethasone (7). CD38 is a transmembrane glycoprotein highly expressed on MM cells that acts as both a receptor and an ectoenzyme. It is also involved in the activation and proliferation of immune cells (Morandi et al.). SLAMF7 is a surface glycoprotein receptor expressed on plasma cells (PCs) and on natural killer (NK) cells that is implicated in adhesion to stromal cells and in the activation of NK cell effector function (8).

Both DARA and Elo share the feature to recruit the immune system to enhance cellular cytotoxicity directed against myeloma cells (9). However, Elo acts only through NK cells and its effects are improved in combination with immunomodulatory drugs (IMiDs) as lenalidomide. On the other hand, DARA and the newer isatuximab (Isa) shows a broad spectrum of activity, including ADCC, ADCP, CDC, and possibly direct induction of apoptosis on MM cells. Further, they exhibit promising results even as a single-agent (9). Beyond mAbs against surface molecules, several agents targeting immune checkpoints (e.g., CTLA-4, LAG3, PD-1/PD-L1, ICOS) expressed on immune cells have also been recently developed as a therapeutic strategy to activate T-cell mediated anti-tumor immunity (10). Specifically, the PD-1/PD-L1 axis has emerged as a central immune checkpoint that controls anti-tumor immune responses and plays a critical role in the metabolic reprogramming of cancer cells within solid tumors. However, its role in MM progression remains to be clarified. Discordant results have been reported on PD-1/PD-L1 expression in MM thus suggesting the need of a more precise definition of PD-1/PD-L1 distribution in the context of cells within the MM tumor microenvironment (Costa et al.). Interestingly, the expression of immunecheckpoint molecules by osteoclast has been recently underlined. However, single-agent studies on PD-1/PD-L1 inhibitors have not demonstrated significant responses in MM patients. On the other hand, other studies have demonstrated the ability of lenalidomide to enhance anti-MM immune activity mediated by PD-1/PD-L1 inhibition despite high grade of toxicity (Jelinek et al.).

The use of mAbs in therapy now led to the observation of antibody resistance, which may appear at different times. Different approaches were designed in order to answer this issue, which is of critical relevance in clinics. Hypothesis or observations explaining the effects may be referred to down modulation of the target molecule by the neoplastic cells. An alternative is represented by a re-distribution of the target molecule on selected surface domains (e.g., polar aggregation or capping). Polar aggregation tends to coalescence the CD38 molecule with ectoenzymes involved in the production of adenosine along with inhibitory complement receptors (CD46, CD55, and CD59) and PD-L1. The availability now of a second antibody with the same the same specificity but recognizing a different epitope may be proposed when the resistance to the first one is observed. This strategy is expected to bypass the resistance mechanisms and to exert new mechanisms of therapeutic action.

## Trying to Design the Future

The design of innovative strategies in MM therapy is a difficult challenge, since the disease has been adopted as a model where different immunotherapeutic approaches are under evaluation. For these reasons, we would like to focus to some aspects, sometime not considered to design the future of the immunotherapy in MM. Most details and complete authoritative reviews may be found in the manuscripts of this Special Issue.

1. *New target markers for mAb therapy*. The efforts to identify specific markers exclusively identifying human myeloma cell surface has been quite disappointing. So far, a criteria adopted is quantitative (e.g., for anti-CD38) or based on clear receptorial features (e.g., for CS1 or SLAMF7). B cell maturation antigen (BCMA) was recently adopted in virtue of a quite restricted expression, along with APRIL, one of its ligand. All the potential targets for antibody-mediated therapy are summarized in a recent and complete reviews (11, 12).
2. *Cell-based therapies*. Beside the mAb-mediated approaches alone or as drug carriers or bispecific T cell engager, the cellular approaches using genetically modified T lymphocytes (CAR-T) or NK cells are expanding exponentially and analyzed (11). Such approaches are reviewed in papers of the Special Issue.
3. *Extension of NK cell life and activity*. Strategies to extend the life and performance of NK cells is one of the hot areas in the field. Paiva group analyzed the gene profile obtained in NK cells exposed to Isa: among the up-modulated appeared CD137, an inducible molecule (also known as Tumor Necrosis Factor Receptor Super Family-9, TNFRSF-9) (13). For this molecule, there are available two different antibodies, used for clinical trials. Their use combined with Isa aimed at increasing the life span of NK cells produced unsatisfactory results, at least in the model adopted (13). However, new observations support the possibility of combinations between therapeutic anti-CD38 and anti-CD137. The disappointing results obtained *in vivo* with anti-CD137 mAbs (urelumab, a human IgG4, and utomilumab, a human IgG2) were likely attributable to negative effects mediated by their interactions with FcRs (14). Now a construct with an arm made of a recombinant trimetric form of the CD137 Ligand (TNFSF-9) associated to the different tumor-associated molecules leads to an *in situ* activation of CD8 cell co-stimulation, with production of IFN-γ (15, 16).
  In order to generate potent antibodies against tumor cells and stimulating anti-tumor cell immunity, recently, trifunctional natural killer (NK) cell engagers (targeting NKp46 and CD16 on NK cells) and a tumor antigen on cancer cells have been developed. This approach produced *in vitro* more potent effects than the therapeutic antibodies used in clinics to target the same tumor antigens (17).
4. *FcR engagement and effects induced by target ligation*. No systematic analysis of this step was conducted on anti-CD38 therapy to date. It is reasonable to expect that the differences in structure between DARA and Isa (one full human, the other one chimeric mAbs) may be reflected on the interactions with the IgG FcRs. Results obtained *in vitro* giving DARA-armed FcR^+^ cells instead of soluble mAb is followed by a distinct membrane dynamics. Critical here are the effects induced by antibody ligation on the tumor target molecule. It is reported that this event may be followed by internalization of the target/antibody complex or externalization, followed by a release in the biological fluids in the form of microvesicles.
5. *Combination therapies*. Part of actual therapeutic potential of the different type of antibody approach may be improved by using immune modulators or combination with other mAbs (with similar or different specificities) or recombinant constructs. A limit on the use of reagents targeting one or two different molecules (surface targets or modulators of the immune response) comes from the recent evidence that the MM is characterized by a marked spatial genomic heterogeneity, with an early phase with clonal sweeps followed by a regional evolution in advanced stages of the disease (18).

## Author Contributions

NG and FM equally contributed to write the manuscript and made a substantial, direct and intellectual contribution to the work, and approved it for publication.

### Conflict of Interest Statement

NG received research funding and honoraria from Amgen, Bristol Mayers Squibb, Celgene, Millenium Pharmaceutical, and Janssen Pharmaceutical. FM has received honoraria for lectures and participation on the advisory boards of Janssen, Tusk Therapeutics, Takeda, and Sanofi, along with research agreements from Janssen and Tusk Therapeutics. The authors have no other relevant affiliations or financial involvement with any organization or entity with a financial interest in or financial conflict with the subject matter or materials discussed in the manuscript apart from those disclosed.
